# Editorial: Cell Communication in Vascular Biology

**DOI:** 10.3389/fphys.2021.656959

**Published:** 2021-03-03

**Authors:** Mauricio P. Boric, Walter N. Durán, Xavier F. Figueroa

**Affiliations:** ^1^Departamento de Fisiología, Facultad de Ciencias Biológicas, Pontificia Universidad Católica de Chile, Santiago, Chile; ^2^Department of Pharmacology, Physiology and Neuroscience, New Jersey Medical School, Rutgers, The State University of New Jersey, Newark, NJ, United States

**Keywords:** vascular function, blood flow distribution, cell communication, endothelial cell, vascular smooth muscle cell, vascular diseases, cerebral circulation

This Research Topic comprises 5 review articles and 10 original contributions in the field of cell communication in Vascular Biology. Contributors used a variety of experimental approaches and models, or exercised keen analysis of published reports, to assess intercellular signaling between different pairs of cell types, such as the endothelium with vascular smooth muscle (VSM), platelets, leukocytes, tumor cells, and trophoblasts; but also, VSM and macrophages or among complex systems like intact vessels, the neurovascular unit and placenta. Communication includes direct cell-cell contact by adhesion molecules or gap junctions; classic receptor-mediated paracrine signaling and intercellular genetic modification by non-coding RNAs (NcRNA) or microvesicles transfer; all of this in the context of physiological regulation and different pathologies.

Control of blood flow supply by the vascular system is essential to keep the homeostasis of each cell of the organism, and then, blood flow distribution must be dynamically regulated to match the changing metabolic demand of surrounding tissue. Although the amount of blood flow depends on the total cross-sectional area of arteries (i.e., total number and caliber) irrigating a particular territory, blood vessels are not inert conduits aimed to passively transport blood into the tissues, but they actually are complex, multicellular structures that must work as a unit to rapidly adjust the distribution of blood flow according to the minute-to minute cellular requirements (Segal et al., 2000; Segal, 2005). The vessel wall is mainly constituted by smooth muscle cells and endothelial cells; thus, cell-to-cell communication is fundamental for the fine synchronization of function between these two cell types along the length of the vessels (Segal, 2015). It should be noted that synchronization and coordination are accomplished by an intricate system of complementary signaling pathways, which involves the interaction of direct cell-to-cell communication via gap junctions and the release of autocrine/paracrine signals (Figueroa and Duling, 2009; Moncada and Higgs, 2018). The relevance of this interaction is highlighted by the fine coordination of endothelial and smooth muscle cell function observed in the control of vasomotor tone through gap junctions located at the myoendothelial junctions (i.e., myoendothelial gap junctions) and endothelium-derived relaxing signals, such as nitric oxide (NO), prostaglandins and an unidentified signal known as endothelium-derived hyperpolarizing factor (EDHF) (Busse et al., 2002). The regulation of these signaling pathways is elegantly depicted in this Research Topic by Schmidt and de Wit.

Gap junctions are made up by the association of two hemichannels provided by each adjacent cell and, in turn, hemichannels are formed by connexin proteins (Sáez et al., 2003; Molica et al., 2018). However, individual hemichannels can be functional and work as a complementary signaling pathway to pannexins, which form channels with similar characteristics to hemichannels. Both connexin hemichannels and pannexin channels can contribute to the intercellular vascular signaling by the release of autocrine/paracrine signals, such as ATP, NO, and PG (Begandt et al., 2017), as noted in the article of Kameritsch and Pogoda.

Coordination of endothelial and smooth muscle cell function does not only rely on the intercellular signaling generated in a particular vessel segment (i.e., radial communication), but also on the longitudinal communication along the whole vessel length. In addition, small arteries and arterioles form a complex network in the microcirculation, where different vessel segments must work in concert to regulate blood flow distribution by precise, well-integrated changes in the luminal diameter (Segal, 2005; Figueroa and Duling, 2009), as demonstrated by the conduction of vasomotor responses along the longitudinal axis of microvessels (Møller et al.).

In addition to the complex signaling interaction between the cells of the vascular wall, regulation of blood flow distribution in peripheral tissues depends on both local signals from the surrounding tissues and the autonomic nervous system, basically the sympathetic nervous system (Thomas and Segal, 2004; Westcott and Segal, 2013; Gaete et al., 2014). However, paradoxically, cerebral blood flow is mainly controlled by local signaling across the neurovascular unit (neurons, astrocytes and cell types of the vascular wall) through a communication mechanism known as neurovascular coupling or vasculo-neuronal coupling, depending on the direction of the signaling, from neurons to vessels or from vascular cells to neurons, respectively (Moore and Cao, 2008; Muñoz et al., 2015; Kim et al., 2016; Iadecola, 2017). Importantly, the article of Presa et al. illustrates that pathological conditions, such as hypertension, may lead to alterations in these communication mechanisms with a subsequent cognitive decline.

Not only the amount of blood flow through different territories must be regulated according to demand, but the vascular wall constitutes a regulated barrier for transport or exclusion of small metabolites, proteins and circulating blood cells. In this context, brain function also relies on the control of neuronal environment by endothelial cells in the blood brain barrier (BBB) (Obermeier et al., 2013) and the functional integrity of the barrier can be stressed in inflammatory conditions, as shown by Ittner et al. It should be noted that endothelial cells are not only important for the correct BBB function, but also for the blood-labyrinth-barrier (BLB) integrity of the stria vascularis in the cochlea (Shi, 2016). Interestingly, connexin-mediated communication plays a central role in the BBB signaling (Gaete et al., 2014) and now the study of Zhang J. et al. extends these data, demonstrating the relevance of Cx43 in the BLB functional integrity and, consequently, in the hearing function. In addition to the regulation of endothelial barrier integrity and exchange functions, cell adhesion and transmigration are important features that may be altered in pathological conditions (Durán et al., 2010; Filippi, 2019). The mechanisms involved in the communication of the vascular wall with blood circulating cells are complex; and in this article collection, endothelial interaction with and transmigration of leucocytes and tumor cells in the peripheral circulation, and the role of NO in these processes is clearly detailed by Aguilar et al..

Two complementary compartments can be recognized in the vascular system, the arterial and venous circulations. Both compartments, in turn, are divided into two functionally different vascular territories: the macrocirculation and microcirculation, which are structurally designed to perform different functions in response to distinct stimuli. In this context, it is noteworthy that fine control of gene expression is essential to maintain the functional integrity of the different segments of the vascular system and, consistent with this notion, the progress of diverse vascular-related pathologies is associated with variations in the transcriptional profile (Lamont and Childs, 2006; Man et al., 2016). Therefore, a thorough analysis of the changes in the gene expression pattern is required to understand the morphological and functional modifications observed in diverse pathological and clinical conditions, which highlights the contribution of the studies reported in this article collection regarding vessel remodeling induced by drastic hemodynamic changes, by Zhang X. et al. in an aortic constriction model and Jie et al. in an arteriovenous fistula model. Likewise, in agreement with the relevance of the fine control of gene expression in vascular homeostasis, regulatory NcRNA, such as long non-coding RNAs (lncRNA) and microRNAs (miRNA), have been recognized to be key players in the control of endothelial and smooth muscle cell function (Balamurali and Stoll, 2020; Ono et al., 2020). Accordingly, NcRNAs have been found to be involved in the pathogenesis of several cardiovascular diseases, but also, they have been proposed as therapeutic target molecules, in line with the results presented in the articles of Cheng et al. in spine cord injury repair and Su et al. in diabetic neointimal hyperplasia. Interestingly, miRNAs can be transmitted through microvesicles (Van Niel et al., 2018), which is addressed in this article collection by Zheng et al., showing that miRNA release via endothelial microvesicles can be involved in the pulmonary vascular leakage and lung injury observed in sepsis.

Cardiovascular diseases are the leading cause of morbidity and mortality worldwide and endothelial cells play a fundamental role in the control of cardiovascular homeostasis by the synthesis and release of a wide spectrum of signals that modulate angiogenesis, inflammation, hemostasis, vasomotor tone, and vascular permeability (Sturtzel, 2017). Then, in agreement with the importance of the endothelium, disturbance, impairment, or loss of normal function of endothelial cells (i.e., endothelial dysfunction) has been recognized as one of the most prominent risk factors for the development of cardiovascular diseases (Endemann and Schiffrin, 2004; Hirase and Node, 2012). Endothelial dysfunction has been directly linked with the initiation and evolution of hypertension and atherosclerosis (Gimbrone and García-Cardeña, 2016; Konukoglu and Uzun, 2016), but it has also been associated with physiological as well as pathophysiological processes, including aging, heart failure, coronary syndrome, thrombosis, intravascular coagulation, type I and II diabetes, obesity, inflammation, sepsis, and sleep apnea syndrome (Endemann and Schiffrin, 2004). In this article collection, the role of endothelial dysfunction in pre-eclampsia is highlighted and strengthened by the work of Liu et al.. However, it should be noted that vascular homeostasis and integrity also depend on the smooth muscle layer of the vessel wall (Majesky, 2016); in this context, Beck et al. address the impact of endothelial dysfunction in arterial remodeling in diabetes. Similarly, Ackers et al. show that alterations in smooth muscle cell signaling pathways can lead to the development of vascular diseases, such as atherosclerosis.

Overall, this Research Topic provides a comprehensive review and original contributions for the continued investigation of regulation of cell communications in vascular biology.

## Author Contributions

XF: manuscript writing and edition. MB and WD: contribution in manuscript writing and edition. All authors contributed to the article and approved the submitted version.

## Conflict of Interest

The authors declare that the research was conducted in the absence of any commercial or financial relationships that could be construed as a potential conflict of interest.
